# Biomarker pathway heterogeneity of amyloid‐positive individuals

**DOI:** 10.1002/alz.14287

**Published:** 2024-10-17

**Authors:** Lloyd Prosser, Carole H. Sudre, Neil P. Oxtoby, Alexandra L. Young, Ian B. Malone, Emily N. Manning, Hugh Pemberton, Phoebe Walsh, Frederik Barkhof, Geert Jan Biessels, David M. Cash, Josephine Barnes, for the Alzheimer's Disease Neuroimaging Initiative

**Affiliations:** ^1^ Dementia Research Centre Department of Neurodegenerative Disease UCL Queen Square Institute of Neurology Queen Square London UK; ^2^ School of Biomedical Engineering and Imaging Sciences King's College London London UK; ^3^ Centre for Medical Image Computing University College London London UK; ^4^ MRC Unit for Lifelong Health and Ageing at UCL Department of Population Sciences and Experimental Medicine University College London London UK; ^5^ Department of Radiology and Nuclear Medicine VU University Medical Center Amsterdam Neuroscience Amsterdam the Netherlands; ^6^ Department of Neurology and Neurosurgery UMC Utrecht Brain Center University Medical Center Utrecht Utrecht the Netherlands

**Keywords:** Alzheimer's disease, amyloid positive, heterogeneity, mixed dementia, subtype and stage inference, vascular pathology

## Abstract

**INTRODUCTION:**

In amyloid‐positive individuals, disease‐related biomarker heterogeneity is understudied.

**METHODS:**

We used Subtype and Stage Inference (SuStaIn) to identify data‐driven subtypes among cerebrospinal fluid (CSF) amyloid beta (1‐42)–positive individuals from the Alzheimer's Disease Neuroimaging Initiative (ADNIGO/2 [*n *= 376]). Variables included: CSF phosphorylated tau (p‐tau181), hippocampal and whole‐brain volume, logical memory (LM), composite Trail Making Test score, and white matter hyperintensity (WMH) volumes. CSF amyloid‐negative, apolipoprotein E ε4 non‐carrier cognitively unimpaired controls (*n *= 86) were used to calculate *z* scores.

**RESULTS:**

One subtype (*n *= 145) had early LM changes, with later p‐tau and WMH changes. A second subtype (*n *= 88) had early WMH changes, were older, and more hypertensive. A third subtype (*n *= 100) had early p‐tau changes, and reflected typical Alzheimer's disease. Some amyloid positive (*n *= 43) individuals were similar to the amyloid‐negative group.

**DISCUSSION:**

This work identified heterogeneity in individuals who are conventionally considered homogeneous, which is likely driven by co‐pathologies including cerebrovascular disease.

**Highlights:**

- Data‐driven modeling identified marker heterogeneity in amyloid‐positive individuals.
- Heterogeneity reflected Alzheimer's disease‐like, vascular‐like, and mixed pathology presentations.
- Some amyloid‐positive individuals were more similar to amyloid‐negative controls.
- Vascular pathology plays a key role in understanding heterogeneity in those on the amyloid pathway.

## 1 INTRODUCTION

The National Institute on Aging–Alzheimer's Association research framework suggests that the detection of amyloid beta (Aβ) 1‐42 (amyloid) is sufficient as a biological definition of the Alzheimer's disease (AD) continuum,^1^ with amyloid abnormality (measured either by cerebrospinal fluid [CSF] or positron emission tomography [PET]) preceding other AD manifestations.^2^

Following the amyloid cascade hypothesis,^3^ hypothetical models have proposed a temporal ordering of biomarkers prior to symptom development.^1^ In these, first amyloid deposition occurs, followed by accumulation of intracellular tangles comprised of tau protein, then brain atrophy, and finally worsening memory and daily functioning. While this provides some understanding of the expected biomarker ordering pathway, there is likely some heterogeneity in the ordering among groups of individuals. One study exploring the temporal ordering of biomarkers in a combined group of cognitively unimpaired individuals, those with mild cognitive impairment (MCI), and those with AD, derived heterogeneous subtypes displaying a typical AD pathway, alongside others displaying a more vascular or mixed pathology temporal ordering.^4^ Heterogeneity was further explored by Habes et al.,^5^ and identified atrophy pattern differences (hippocampal sparing, medial temporal lobe dominant, parietal dominant, limbic predominant) in an AD‐like cohort.

Other factors like those mentioned above contribute to the heterogeneity of disease, meaning homogeneity of those already on the amyloid pathway is unlikely. As potential therapies target removal of amyloid deposition,^6^ having a clear understanding of the possible progression pathways that individuals may follow would allow better prediction of potential trajectories in studies and assessments.

Known examples of heterogeneity in amyloid‐positive individuals include a subtype in which there is minimal atrophy in the hippocampus while general cerebral atrophy is present.^7^ Meanwhile there is evidence that although individuals may be amyloid positive, they do not necessarily further progress clinically.^8^ Heterogeneity may also be driven by the presence of vascular pathology, with white matter hyperintensities (WMH) being a key marker related to future clinical progression.^9^ WMH pathology seems to have an additive relationship with Aβ,^10^ accelerating the progression of AD.

As AD processes likely occur over decades, long‐term follow‐up of individuals would be ideal to identify different groupings of disease trajectories. However, such studies are expensive and prone to substantial drop‐out. Recent data‐driven methods allowed identification of heterogeneity in biomarker ordering using cross‐sectional data. One such data‐driven method, Subtype and Stage Inference (SuStaIn) can be used to simultaneously subtype and stage individuals using cross‐sectional data,^11^ and has been applied in multiple diseases and is validated longitudinally.^12^, ^13^, ^14^

This study applied SuStaIn to amyloid‐positive individuals, to discover data‐driven subtypes of those on the amyloid pathway. Here we look at typical markers associated with AD (cognitive impairment, tau, and brain volumes), along with a marker of presumed cerebrovascular disease (WMH). It was hypothesized that one subtype would follow the typically identified pattern (tau, then atrophy, then cognitive scores), whereas another subtype might show early presumed cerebrovascular disease. It is also hypothesized that, despite these individuals having evidence of amyloid deposition, a group like cognitively unimpaired controls would be identified.

## 2 METHODS

### 2.1 Cohort

Data used in the preparation of this article were obtained from the Alzheimer's Disease Neuroimaging Initiative (ADNI) database (adni.loni.usc.edu). The ADNI was launched in 2003 as a public–private partnership, led by Principal Investigator Michael W. Weiner, MD. The primary goal of ADNI has been to test whether serial magnetic resonance imaging (MRI), PET, other biological markers, and clinical and neuropsychological assessments can be combined to measure the progression of MCI and early AD. For up‐to‐date information, see www.adni‐info.org.

Newly enrolled ADNI2 and ADNIGO individuals were used in the current study. This included (1) a CSF amyloid‐negative, cognitively normal (CN) diagnostically stable group (amyloid‐negative controls) and (2) a group of amyloid‐positive cases.

The amyloid‐negative controls were used to produce normalized *z* scores of the amyloid‐positive cases. To be included in this group, controls had to be CSF amyloid negative (above cut‐point 262 pg/mL), apolipoprotein E (*APOE*) ε4 non‐carriers, and have remained diagnostically CN throughout their follow‐up in ADNI.

**RESEARCH IN CONTEXT (120/150)**

1. **Systematic review**: Authors reviewed literature using traditional (e.g., PubMed) sources. We assessed articles that examined Alzheimer's disease (AD)–related and vascular marker heterogeneity in amyloid‐positive individuals. This included associations between markers, and potential temporal orderings of marker abnormalities.
2. **Interpretation**: We found that three heterogenous subtypes were present in a cohort of amyloid‐positive individuals. This included a group of individuals with a typical AD‐like pathway, a more mixed pathology group, and a more vascular‐dominant group. We add to the continued literature that emphasizes the likely important role of vascular pathologies in AD.
3. **Future directions**: As the role of vascular pathologies becomes more present in AD, understanding how this further reflects temporal and continued heterogeneity in AD‐assumed cohorts is necessary.

The amyloid‐positive cases were those used to create data‐driven SuStaIn subtypes. This group was defined as CSF amyloid positive (below cut‐point 262 pg/mL). Individuals could have any diagnosis at baseline or follow‐up.

CN individuals were defined by having a Mini‐Mental State Examination (MMSE) score between 24 and 30 (inclusive) at baseline and a Clinical Dementia Rating (CDR) score of 0. CN individuals were normally functioning as measured by education‐adjusted scores on delayed recall of one paragraph from Wechsler Memory Scale Logical Memory II. CN individuals who reported subjective memory concerns were labelled SMC. Individuals with MCI were required to have an MMSE score between 24 and 30 (inclusive) at baseline, objective memory loss by education‐adjusted scores on Wechsler Memory Scale Logical Memory II, a global CDR equal to 0.5, and report subjective memory concern. Individuals with AD were defined by having an MMSE score between 20 and 26 (inclusive), a CDR of 0.5 or 1.0, subjective memory concern, and National Institute of Neurological and Communicative Disorders and Stroke–Alzheimer's Disease and Related Dementias Association criteria for probable AD.

Individuals were given a diagnosis at baseline, month 6, month 12, and then yearly. At follow‐up, those with evidence of clinical progression were given a converting diagnosis by a physician on site, whereas those with improvements may receive a reverting diagnosis.

To be included in this study, individuals had to have a complete set of observations of CSF Aβ1‐42 and phosphorylated tau (p‐tau181) at their baseline visit; suitable MRI scans that produced quality measures of WMH, whole‐brain, hippocampal, and total intracranial volume (TIV) measurements; and neuropsychology test scores for Trail Making Test (TMT) A, TMT B, and logical memory (LM).

### 2.2 CSF measurements

Baseline CSF Aβ1‐42 and p‐tau181 measurements (raw) from the ADNI biomarker core (University of Pennsylvania) using the microbead‐based multiplex immunoassay, the INNO‐BIA AlzBio3 RUO test (Fujirebio, Ghent, Belgium), on the Luminex platform (LuminesCorp, Austin, TX, USA) were obtained (UPENN‐CSF‐Biomarker‐Data‐Master [ADNI1, GO, 2], Version: 2016‐07‐05).

A Gaussian mixture model (GMM) established the value of CSF amyloid used to identify amyloid‐negative and amyloid‐positive individuals, using available baseline CSF amyloid raw values for all ADNI2/GO individuals. In this, a histogram was plotted for the data and two data‐driven bimodal Gaussians were identified. A cut‐point was used to separate the two Gaussians at 99th percentile.

### 2.3 Cerebrovascular measurements

WMH of presumed vascular origin were previously calculated using cross‐sectional Bayesian model selection (BaMoS) applied to T2 fluid‐attenuated inversion recovery (FLAIR) and T1‐weighted images,^15^ with all outputs visually assessed by experienced raters.^16^ Numbers of probable and definite microbleeds were previously identified and counted using the Microbleed Anatomical Rating Scale (MARS).^17^

The number of lacunes of presumed vascular origin were obtained from measures in previous work by our group.^4^ Lacunes were identified on T2‐FLAIR, using co‐registered T1‐weighted imaging as an anatomical reference. Lacunes were only included in regions of white matter in the territory of perforating arterioles and of size between 3 and 15 mm. All lacunes were checked by a neuroradiologist.

### 2.4 Brain volume measurements

Whole‐brain, hippocampal, and total intracranial volume (TIV) were previously extracted from T1‐weighted scans. Whole‐brain volumes were calculated using the automated Multi‐Atlas Propagation and Segmentation (MAPS) tool,^18^ with quality control and manual edits made using MIDAS.^19^ Hippocampal volumes were obtained using a similar approach, using Similarity and Truth Estimation for Propagated Segmentations (STEPS),^20^ and TIVs were calculated from T1‐weighted images using geodesic information flows (GIF).^21^

### 2.5 Neuropsychology

LM and TMT A and TMT B were acquired as part of ADNI neuropsychology battery. LM scoring was based on total number of story units recalled. Both TMT A and TMT B are scored as time to complete. For TMT B, a ceiling is present for individuals that took longer than 300 seconds to complete the task. A composite TMT score was produced, as scores are correlated, and TMT A and B are argued to be a less specific measure of executive functioning alone. A more specific measure of executive functioning was derived using TMT B minus TMT A^22^, as it is suggested to minimize visuoperceptual and working memory demands.

### 2.6 Neurofilament light

Neurofilament light (NfL) quantification was performed by Blennow labs and downloaded from ADNI. Plasma NfL was analyzed using the single molecule array (Simoa) technique. The assay used a combination of monoclonal antibodies and purified bovine NfL as a calibrator.^23^ Samples were measured in singlicate, with a technical lower limit 6.7 pg/mL.

### 2.7 Demographics, *APOE*, and medical history

Diagnostic and demographic data (age, sex, *APOE* ε4 carrier status, medical history [hypertension and stroke]), and follow‐up time were downloaded from the ADNI database (adni.loni.usc.edu/).

### 2.8 Statistical analysis

#### 2.8.1 Data transformation

WMH were log transformed (log_2_). As SuStaIn regards increasing numbers as a poorer score, negative direction scores (LM, hippocampal volume, whole‐brain volume) were inverted.

Using the amyloid‐negative controls, *z* scores were produced for LM, TMT composite, and p‐tau. For WMH, whole‐brain volume, and hippocampal volume, covariate‐corrected *z* scores were produced to account for TIV.^24^

#### 2.8.2 SuStaIn

A *z* score SuStaIn approach was followed, and is explained in previous work^11^ Briefly, *z* score SuStaIn is a generalization of the original event‐based model.^25^, ^26^ The event‐based model describes disease progression as a series of events, with each event corresponding to biomarker progression from normal to abnormal. The *z* score model of SuStaIn follows from this approach with each event representing the linear accumulation of biomarkers from one *z* score (1 standard deviation difference from amyloid‐negative control group mean) to another. This results in variable patterns of *z* score events, producing heterogeneous groups (subtypes).

To be included in the SuStaIn model, *z* scores from continues markers were needed. Here we look at typical markers associated with AD (cognitive impairment, tau, and brain volumes), along with a marker of presumed cerebrovascular disease (WMH) as cerebrovascular disease and dysfunction has been shown to be a core feature of AD.^27^, ^28^ The markers of interest included in the current model assess features anticipated to become abnormal in those progressing along the amyloid pathway according to the hypothetical model of AD. This includes cognition (LM, composite TMT); tau (CSF p‐tau181); neurodegeneration (whole‐brain volume and hippocampal volumes), along with a measure of presumed cerebrovascular disease (WMH volume). As lacunes and cerebral microbleeds (CMBs) were binary measures, they were not included in the SuStaIn model, but were included in demographics.

This implementation of SuStaIn used *z* scores 1 through 3, and had a maximum number of *z* scores of 5. Histograms of the *z* scores were produced to visualize that three *z* scores was appropriate to represent the data, with three *z* scores representing ≈ 75% of the data (Figure S1 in supporting information).

The log likelihood across Markov chain Monte Carlo (MCMC) samples were initially used, across each subtype. Separation of MCMC trace suggests distinctly different subtypes. Ten‐fold cross‐validation was performed to further investigate the optimal number of subtypes within the data. The cross‐validation information criterion (CVIC) is an information criterion that balances model complexity with model accuracy, with a lower CVIC indicating a better balance between the two. Models with the lowest CVIC would be the better fit; however, small improvements (< ≈ 6) in CVIC with a more complex model would suggest use of a less complex (fewer subtyped) model. A maximum of four subtypes were tested, with the best balance from MCMC and CVIC found using three subtypes (see Figures S2, S3 in supporting information). Sample sizes were also plotted as a histogram in Figure S4 in supporting information, with smaller sample sizes in later stages suggesting higher stage uncertainty. Probabilities over SuStaIn stages were plotted in Figure S5 in supporting information, to ensure no crossover events are present in each stage.

#### 2.8.3 Evaluation of cohort

We provided demographic details of the entire cohort by their baseline diagnosis, prior to subtyping using SuStaIn. This included markers used in the SuStaIn model, and other markers of interest.[Table alz14287-tbl-0001]

#### 2.8.4 Evaluation of identified subtypes

Tables and figures were produced to better explain the data‐driven subtypes, and differences between groups are described. This included reporting demographics, genetics, imaging markers, and medical history, with linear regression and Fisher exact test. We also present mean and standard deviations for SuStaIn markers after subtypes were derived. Later diagnostic progression of individuals (from diagnostic CN, or MCI) within their subtype, is also reported with Fisher exact test used to evaluate proportional difference between subtypes.

#### 2.8.5 Visualization of results

Subtypes are plotted in a positional variance diagram to interpret subtype progression. This includes the staging of the event by *z* score distance away from the normalized amyloid‐negative controls (one *z* score [red], two *z* scores [magenta], three *z* scores [blue]). We refer to this as a *change* or *event* throughout this work. Colors between these three *z* scores represent uncertainty in the event staging. In later stages with smaller sample sizes Figure S4), we include a dashed line in each positional variance diagram,^13^ starting from instances with two consecutive stages of two subjects or fewer.

To accompany mean values of SuStaIn markers in each subtype, we also presented individual marker values per SuStaIn stage within each subtype, and report pairwise correlation coefficients to examine relationships of marker difference between event stage within subtype. We report semi‐partial correlations for composite TMT (adjusted for a ceiling effect of TMT B), and for WMH, whole‐brain, and hippocampal volumes (TIV adjusted). We further produced pie charts for each subtype based on their baseline diagnosis, and later diagnostic progression (with Fisher exact test to formally examine these proportions).

## 3 RESULTS

Of an initial sample of 649, 187 were excluded as they did not meet criteria for the amyloid‐negative controls (CSF amyloid‐negative, *APOE* ε4 non‐carrier, stable CN) or amyloid‐positive cases (CSF amyloid‐positive). A further 24 were excluded due to missing marker data (TMT B). This is visualized in Figure S6 in supporting information.

The remaining 462 individuals (amyloid‐negative controls *n* = 86, amyloid‐positive cases *n* = 376) were included in the study.

Table 1 details the demographics of the amyloid‐negative controls and amyloid‐positive cases. Descriptively, the amyloid‐negative controls were on average younger, had higher cognitive scores (composite TMT, LM), lower p‐tau, lower WMH, lower proportions of CMB, and higher whole‐brain and hippocampal volume than the amyloid‐positive cases. Within the amyloid‐positive cases, cognitive scores were lower, hippocampal volume was lower, and p‐tau levels were higher, with diagnostic progression. WMH burden was highest in AD, while CMB proportions were similar between diagnostic subgroups of the amyloid‐positive cases.

**TABLE 1 alz14287-tbl-0001:** SuStaIn cohort basic demographic data.

		Amyloid‐positive cases		
	Amyloid‐negative controls	CN	MCI	AD^*^
*N*#	86	79	220	77
Age at baseline, years	72.4 (5.2)	75.3 (6.4)	72.7 (7.1)	74.6 (8.1)
Sex, *N*# male (%)	50 (58)	31 (39)	123 (56)	42 (55)
First assessment MMSE	29.0 (1.3)	29.0 (1.2)	27.8 (1.9)	23.2 (2.0)
First assessment TMT A	31.4 (10.0)	36.2 (11.0)	40.8 (15.8)	59.4 (33.9)
First assessment TMT B	79.3 (45.3)	92.5 (46.4)	112.1 (57.7)	197.5 (85.8)
First assessment composite TMT	47.8 (43.6)	56.2 (43.2)	71.3 (49.9)	138.2 (70.3)
First assessment LM	14.6 (2.6)	14.1 (3.5)	8.9 (3.4)	4.1 (2.4)
CSF amyloid beta, pg/mL	343.0 (50.7)	194.8 (40.8)	184.7 (39.0)	165.2 (32.5)
CSF ptau181, pg/mL	18.8 (7.4)	26.2 (13.0)	30.2 (13.5)	34.5 (16.3)
WMH median^**^, mL (IQR, mL)	2.5 (3.2)	5.1 (8.8)	4.6 (8.4)	6.1 (8.9)
CMB, N# present (%)	12 (14)	15 (19)	37 (17)	15 (19)
Whole‐brain volume^**^, mL	1091.4 (105.4)	1062.6 (98.6)	1074.4 (106.3)	1024.2 (115.6)
Hippocampal volume^**^, mL	5.6 (0.7)	5.4 (0.6)	5.2 (0.7)	4.6 (0.7)
TIV, mL	1433.0 (136.9)	1405.5 (129.5)	1435.4 (133.1)	1416.3 (150.9)

*Note*: Basic demographics for both the controls and cases used in SuStaIn. Mean values and SD are reported, unless otherwise stated.

Abbreviations: AD, Alzheimer's disease; CMB, cerebral microbleed; CN, cognitively normal/healthy control; CSF, cerebrospinal fluid; IQR, interquartile range; LM, logical memory; MCI, mild cognitive impairment; MMSE, Mini‐Mental State Examination; *N*#, number; p‐tau, phosphorylated tau; SD, standard deviation; SuStaIn, subtype and stage inference; TIV, total intracranial volume; TMT, Trail Making Test; WMH, white matter hyperintensities.

* Probable AD.

** Volumes report in the table are unadjusted.

**FIGURE 1:**
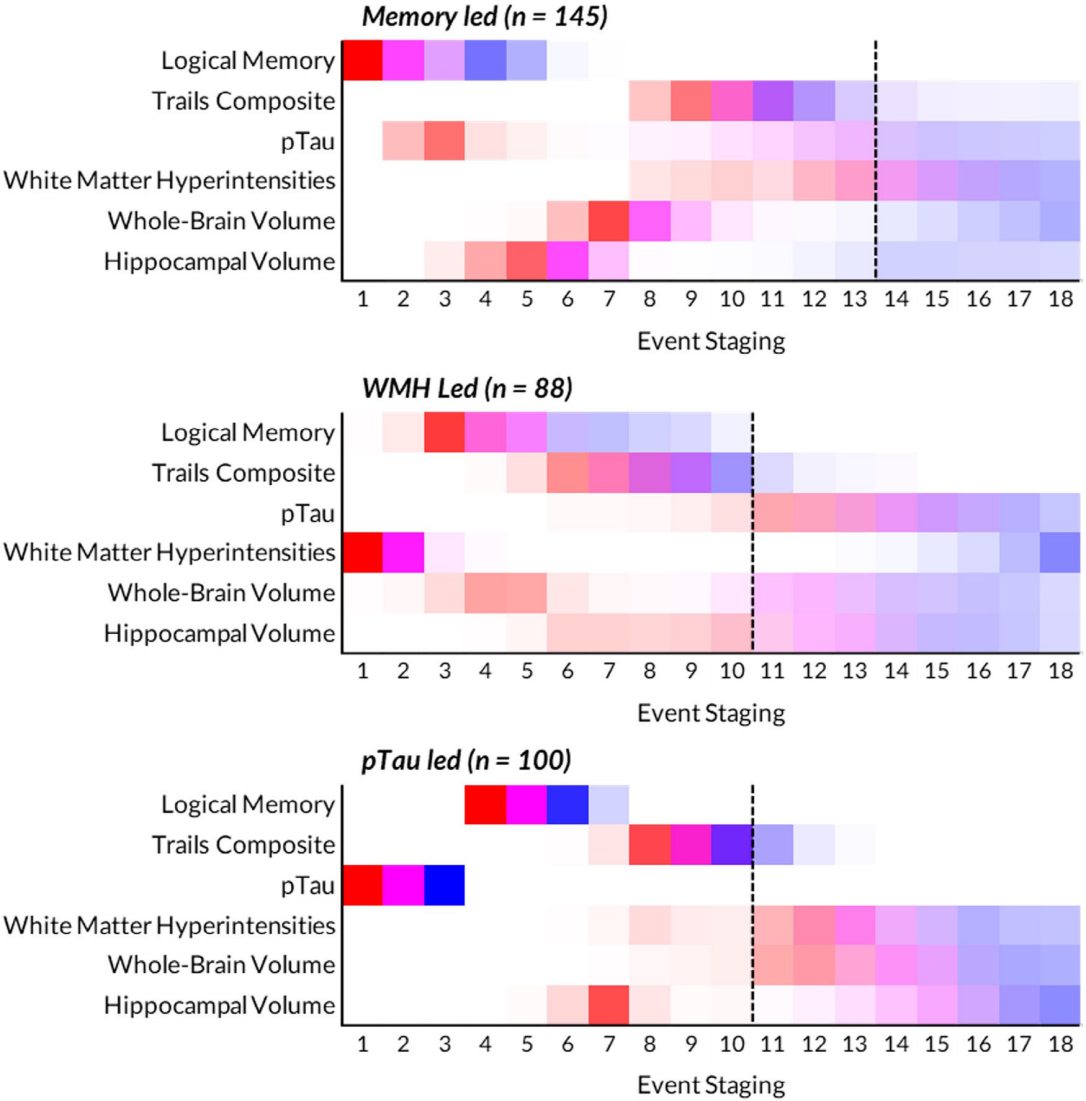
SuStaIn subtyping depicting biomarker event staging by ordering of standardized scores (one *z* score [red], two *z* scores [magenta], three *z* scores [blue]), with colors between these scales highlighting uncertainty in event staging. WMH used log_2_. A dashed line for each subtype is included to represent higher uncertainty in the diagrams, in which number of subjects is ≤ 2 for two consecutive stages. pTau, phosphorylated tau; SuStaIn, subtype and stage inference; WMH, white matter hyperintensities.

From *z* score SuStaIn, three distinct subtypes were derived (see Figure 1 for subtype proportional variance diagrams). A portion of individuals were not subtyped as they did not have different measures to the stable CN group (*n* = 43). We refer to this group as unsubtyped. For subtype one (*n* = 145), named Memory led, SuStaIn determined LM change to be the initial event, followed by p‐tau, hippocampal volume, and whole‐brain volume. For subtype two (*n* = 88), named WMH led, SuStaIn determined WMH change as an initial event, followed by LM, whole‐brain volume, and composite TMT. For subtype three (*n* = 100), named p‐tau Led, SuStaIn determined p‐tau change as an initial event, followed by LM, hippocampal volume, and composite TMT. Event uncertainty is seen in all subtypes, notably in stages ≥ 10, and is characterized by color spread toward later stages.

SuStaIn‐determined subtype demographics are reported in Table 2. The unsubtyped group (*n *= 43) were younger (*P *< 0.001), with a high proportion of CN individuals (53%; two tailed *P *< 0.001). This group also has, on average, more years in education (*p* < 0.001) and the highest MMSE score (*p* < 0.001). They descriptively have a higher proportion of *APOE* ε4 carriers compared to the WMH led subtype, but lower than the Memory and p‐tau led subtypes. The Memory led subtype (*n *= 145) has a higher proportion of MCI and AD individuals (MCI = 64%, AD = 31%; two tailed *p* < 0.001), and is more male (*P *= 0.003). The WMH led subtype (*n *= 88) is older than other subtypes (*p* < 0.001), and has the highest proportion of individuals with hypertension (two‐tailed *P *= 0.03), and lacunes present (*P *= 0.02).

**TABLE 2:** SuStaIn‐determined subtype demographics, genetics, imaging markers, and medical history.

		Subtyped				
	Unsubtyped	Memory led	WMH led	p‐tau led	*p* value^a^	*p* value^b^
*N*#	43	145	88	100		
Age at baseline, years	69.8 (6.2)	73.8 (6.9)	77.9 (6.7)	71.4 (6.9)	< 0.001	< 0.001
Diagnosis, *N*# CN:MCI:AD (%)	23:20:0 (53:47:0)	7:93:45 (5:64:31)	29:48:11 (33:55:13)	20:59:21 (20:59:21)	< 0.001	< 0.001
Sex, *N*# male (%)	21 (49)	88 (61)	48 (54.6)	39 (39)	0.7	0.003
Education, years	17.1 (2.2)	16.3 (2.7)	15.8 (2.6)	15.6 (2.6)	< 0.001	0.1
First assessment MMSE	29.0 (1.5)	26.5 (2.7)	27.4 (2.5)	26.9 (2.8)	< 0.001	0.02
CMB present, *N*# (%)	5 (12)	20 (14)	20 (23)	22 (22)	0.3	0.1
TIV, mL	1418.9 (117.6)	1450.1 (143.3)	1413.2 (125.3)	1402.4 (139.3)	0.8	0.05
Lacunes present (%)	0	0	4 (5)	2 (2)	1	0.02
*APOE* ε4 carrier (%)	25 (58)	101 (70)	39 (44)	72 (72)	0.5	< 0.001
NfL, pg/mL	31.4 (12.5)	41.3 (16.1)	48.0 (21.4)	42.8 (30.2)	< 0.001	0.1
Hypertension, *N*# (%)	18 (42)	69 (48)	57 (65)	51 (51)	0.2	0.03
Stroke, *N*# (%)	0	0	1 (1)	2 (2)	1	0.2

*Note*: Demographics of SuStaIn‐derived subtypes, and the unsubtyped cohort. *p* values representing linear regression or Fisher exact test (diagnosis, sex, CMB, lacunes, *APOE* ε4 carrier, hypertension, stroke) between unsubtyped and subtyped groups,^a^ and between subtypes.^b^ Mean values and SD are reported unless otherwise stated.

Abbreviations: AD, Alzheimer's disease; *APOE*, apolipoprotein E; CMB, cerebral microbleed; CN, cognitively normal/healthy control; MCI, mild cognitive impairment; MMSE, Mini‐Mental State Examination; N#, number; NfL, neurofilament light; SD, standard deviation; SuStaIn, subtype and stage inference; TIV, total intracranial volume; WMH, white matter hyperintensities.

**FIGURE 2:**
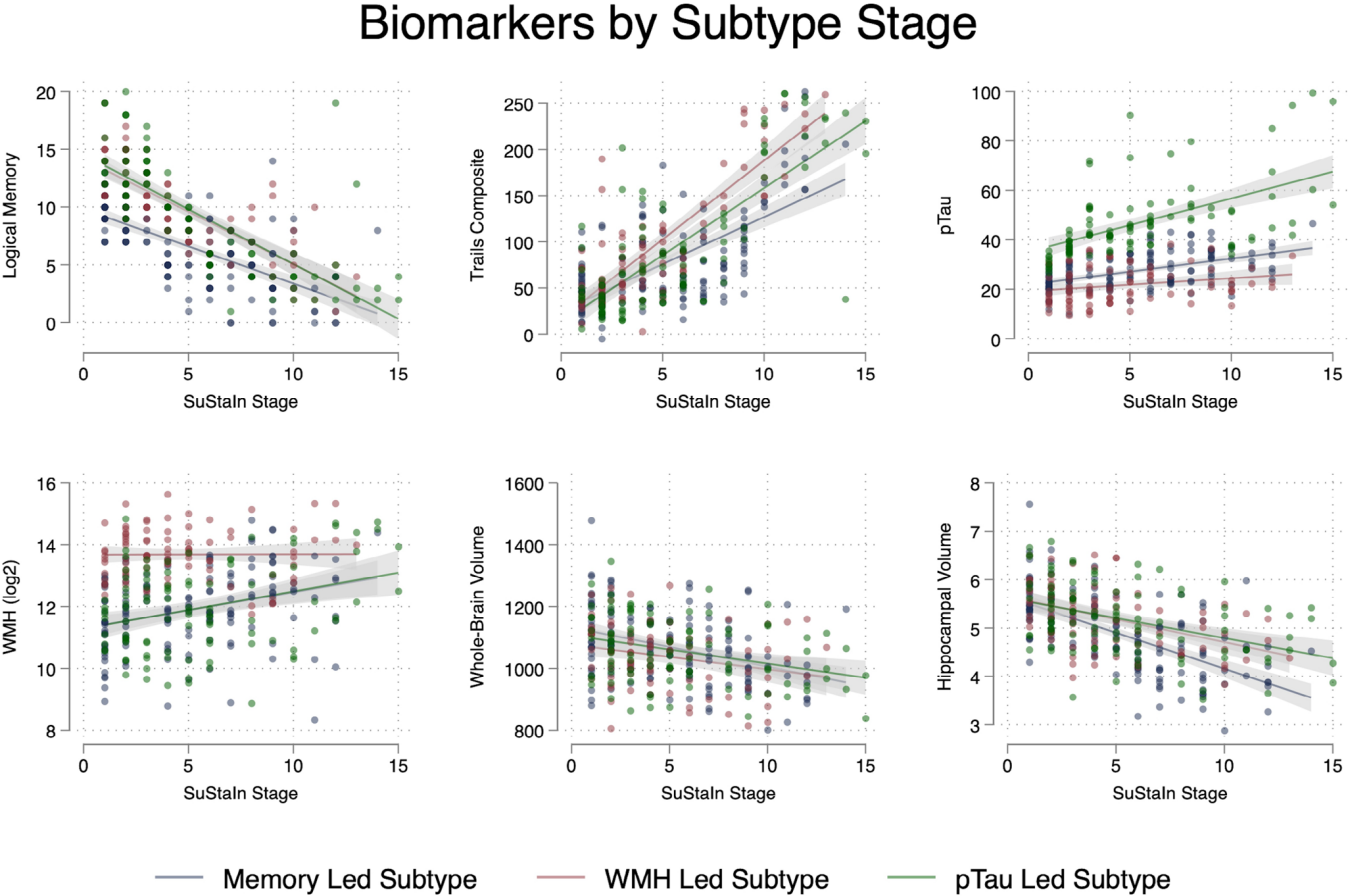
Biomarkers by SuStaIn stage, across subtype. Individual volumes for subtypes for each stage across subtype are plotted, with a line of best fit. Pairwise correlation coefficient across subtype stage is reported in Table S1 in supporting information. pTau, phosphorylated tau; SuStaIn, subtype and stage inference; WMH, white matter hyperintensities.

Mean values for markers used in SuStaIn subtyping are reported in Table 3, with marker level per SuStaIn stage reported in Figure 2 (and Table 1).

**TABLE 3:** Values of markers included in SuStaIn per subtype.

		Subtype		
	Unsubtyped	Memory led	WMH led	p‐tau led
*N*#	43	145	88	100
First assessment LM	14.9 (2.9)	6.5 (3.3)	9.9 (3.7)	9.4 (4.8)
First assessment TMT composite	42.0 (21.9)	77.2 (51.8)	97.0 (64.6)	92.5 (72.0)
CSF p‐tau181, pg/mL	19.0 (5.2)	27.2 (2.5)	21.6 (7.2)	46.9 (14.8)
WMH median^*^, mL (IQR, mL)	2.6 (2.3)	3.8 (4.7)	12.9 (10.3)	3.8 (6.3)
Whole‐brain volume^*^, mL	1095.3 (87.1)	1068.0 (116.1)	1040.1 (101.8)	1057.0 (107.1)
Hippocampal volume^*^, mL	5.6 (0.7)	4.9 (0.8)	5.2 (0.6)	5.2 (0.7)

*Note*: Neuropsychological test scores and CSF/imaging biomarker values per subtype. Mean values and SD are reported unless otherwise stated.

Abbreviations: CSF, cerebrospinal fluid; IQR, interquartile range; LM, logical memory; *N*#, number; p‐tau, phosphorylated tau; SD, standard deviation; TMT, Trail Making Test; WMH, white matter hyperintensities.

* Volumes report in the table are unadjusted.

The unsubtyped group were on average less cognitively impaired (LM, composite TMT), had lower CSF p‐tau, reduced WMH volume, and larger whole‐brain and hippocampal volumes.

The Memory led subtype had the lowest LM score and smallest hippocampal volumes on average. Considering marker difference over SuStaIn stage, there was evidence to suggest significantly lower LM, whole‐brain volume, hippocampal volume, and increased TMT composite, p‐tau, and WMH (*p* < 0.001; all tests).

The WMH led subtype had ≈ 5 mL more WMH volume, lower whole‐brain volumes, and had greatest impairment in composite TMT. Considering marker difference over SuStaIn stage, there was evidence to suggest significantly lower LM, composite TMT, whole‐brain and hippocampal volume (*p* < 0.001; all tests), and a weaker increased p‐tau (pairwise correlation coefficient 0.2, *P *< 0.03). There was no evidence of a difference in WMH volume over event stage.

The p‐tau led subtype had the highest p‐tau values, by > 20 pg/mL. Individuals in this subtype are more likely to be female (*p* < 0.003) and had a higher proportion of *APOE* ε4 carriers (*P *< 0.001) compared to the other subtypes. Considering marker difference over SuStaIn stage, there was evidence to suggest significantly lower LM, whole‐brain volume, hippocampal volume, and increased TMT composite, p‐tau, and WMH (*p* < 0.001; all tests).

**FIGURE 3:**
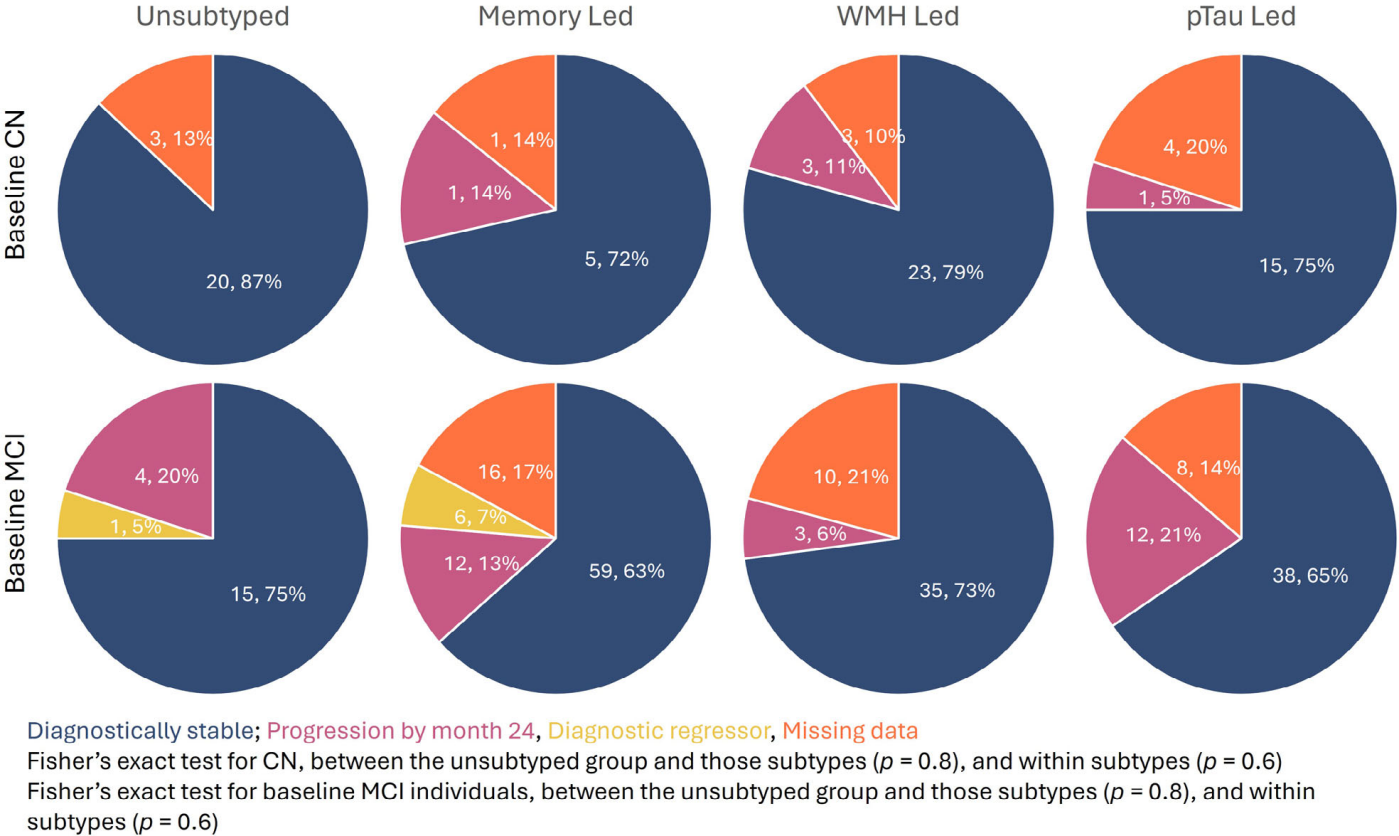
Pie charts for diagnostic progression by 24 months within baseline diagnostic group (CN or MCI), by subtype group. CN, cognitively normal; MCI, mild cognitive impairment; pTau, phosphorylated tau; WMH, white matter hyperintensities.

Results for subsequent diagnostic progression (within 24 months) are displayed in Figure 3. There was no statistical evidence of a difference in progression between the unsubtyped and subtyped groups, or between subtypes. Missing diagnostic data for each subtype is further reported in Table S2 in supporting information.

## 4 DISCUSSION

In this study *z* score SuStaIn was used to investigate biomarker ordering heterogeneity in a cohort of amyloid‐positive individuals. SuStaIn identified three distinct subtypes, and an unsubtyped group.

### 4.1 Memory led subtype

In this subtype (*n* = 145), SuStaIn determined an initial LM event, followed by p‐tau, hippocampal volume, whole‐brain volume, WMH, and composite TMT. Early LM events were likely as most MCI and AD in this cohort were amnestic, with deficits found in MCI^29^ and AD.^30^ The subsequent events of p‐tau, hippocampal, and whole‐brain volume are expected according to the amyloid cascade hypothesis.^1^

CSF p‐tau181 would be expected of those following a conventional AD‐like pathway, although early staging uncertainty may suggest a non‐typical AD pathway.

Earlier hippocampal volume events prior to cognitive domain tasks are typical in those with AD, as associative memory tasks are related to hippocampal activity.^29^ Amyloid deposition increases vulnerability of the hippocampus,^29^ which is a sensitive structure often affected prior to global brain atrophy.^30^ This is supported by outcomes in previous work,^9^ with hippocampal volume a predictor of diagnostic progression. Whole‐brain volume can be considered a general measure of neurodegeneration, and is expected to follow the more specific hippocampal atrophy in those with AD. Like whole‐brain volume, WMH has later stage uncertainty, suggesting vascular pathology may not be as present in this subtype. Composite TMT events were later in this subtype, despite early LM events. This is likely because LM assesses general memory,^31^ while composite TMT assesses executive functioning.^22^

Overall, this subtype seems to represent a mixed AD‐like trajectory, with more cognitive impairment. This is reflective of many AD cases,^32^ particularly after initial diagnosis and at *post mortem*.^33^, ^34^ Although early LM, p‐tau, and hippocampal events suggest an AD‐like group, marker stage uncertainty with p‐tau could suggest other co‐pathologies are present. As pathogenic features in vascular dementia (VaD) and AD^35^ overlap, vascular features are anticipated to influence this group's mixed presentation although lower WMH volume and hypertension risk suggest other underlying co‐pathologies besides cerebrovascular disease may be present. Many with probable AD often show co‐pathologies like TAR DNA‐binding protein 43, α‐synuclein pathology, Lewy bodies, or hippocampal sclerosis at *post mortem*,^36^ and future *post mortem* work must evaluate this.

### 4.2 WMH led

In this subtype (*n* = 88), SuStaIn determined an initial WMH event, followed by LM, whole‐brain, and then hippocampal volume and composite TMT events. This subtype was on average older with higher hypertension risk. Both higher age and hypertension risk are associated with WMH, and may relate to the increased median WMH for the group.^37^ Elevated NfL is likely a non‐specific marker of aging and white matter integrity,^38^ further supporting higher WMH volumes.

Early WMH events (two *z* score) are elevated and remain at similar levels over staging. This group may comprise individuals on a mixed vascular and AD pathway with increased early vascular burden. Given the older age of this cohort, the probability of mixed co‐morbidities is higher than other subtypes.^35^ Similarly to WMH staging, whole‐brain staging has an initial event and then a gap (between stages 6 and 10), which may suggest some variability in neurodegeneration in this group. The later and more uncertain hippocampal volume event staging may suggest minimal hippocampal atrophy. Some literature has suggested that hippocampal volume is reduced in AD populations over VaD,^39^ although WMH does still predict hippocampal volume reduction in those with abnormal amyloid.^40^

The late event of p‐tau, lower mean p‐tau, and lower p‐tau across stages is consistent with literature showing VaD is less affected by p‐tau abnormality.^41^, ^42^ CSF amyloid‐positive individuals with small vessel disease (SVD) have lower cognitive scores and more rapid progression to AD.^43^ Both amyloid abnormality^44^ and increased WMH^45^ are related to cognitive impairment, and seem to be additive.^10^ This may explain why subtype two individuals have earlier cognitive impairment (TMT).

Overall, this subtype seems to represent a population with more SVD burden. They have increased vascular pathology (WMH) and risk factors (hypertension), as well as other indications that separate them from pure AD (low p‐tau, less hippocampal involvement, poorer executive functioning). It is impossible to suggest that this is a diagnostically VaD‐like population, but the similarities to previous literature (reduced hippocampal and p‐tau influence), does suggest that vascular pathologies may be prevalent in this group.

### 4.3 p‐tau led subtype

In this subtype (*n* = 100), SuStaIn determined an initial p‐tau event, followed by LM, hippocampal volume, TMT, and then whole‐brain and WMH volume. This mirrors a typical presentation of those on an AD pathway.^46^ Unlike the other subtypes, there are clear changes (*z* score = 3) for both p‐tau and LM before any changes to other markers included in the model (Figure 2).

The initial and clear event staging of p‐tau would be the expected next stage in the amyloid cascade hypothesis,^46^ and is well correlated with amyloid deposition.^47^ If this is a subtype with extensive tau deposition in the brain, this may influence changes to the hippocampus, which has an event close to that of p‐tau in this subtype. Like the Memory led subtype, earlier lower hippocampal volume events compared to whole‐brain volume is likely due to the hippocampus being specifically affected in AD.^30^ It is one of the first structures to have degenerative change in AD,^48^ and reduced volumes are also present in younger (71 years) individuals with AD compared to healthy counterparts.^49^

The p‐tau led subtype is the youngest subtype. WMH do increase with age,^50^ which may suggest why this subtype has on average lower WMH compared to the WMH led subtype. This group has a lower proportion of hypertension risk (51%), suggesting lower risk of vascular pathology.^51^, ^52^

The early p‐tau events and positive association with stages may suggest this subtype is greatly affected by tau abnormality. This subtype is less affected by vascular risk and disease (WMH, hypertension) and may be a more AD‐like group that follows the pathway outlined in the hypothetical model of AD biomarkers.^46^

### 4.4 Unsubtyped

A proportion (11%) of amyloid‐positive individuals show more similarities to the amyloid‐negative control cases. This may suggest a pre‐symptomatic cohort before clinical decline,^53^ further characterized by the high proportion of CN in this group (53%) although future diagnostic progression within 24 months is not seen (Figure 3). These individuals do have CSF amyloid levels closer to the cut‐point (Figure S7 in supporting information). As CSF amyloid seems to be a more sensitive marker of early change compared to amyloid PET,^54^ these individuals may be at very early stages of a disease pathway that will later accumulate amyloid. This group has a similar proportion of *APOE* ε4 carriers compared to the subtyped groups. This is consistent with previous work that showed increased amyloid positivity in CN *APOE* ε4 carriers,^54^ and a general link between *APOE* ε4 carrier status and amyloid positivity.^8^, ^54^, ^55^

### 4.5 Clinical implications

Identifying a mixed‐pathology group less affected by both p‐tau and other variables or pathologies that may affect decline should be considered. Recognizing alternative trajectories might improve differential diagnosis, better understand future prognosis, and provide better identification of suitable treatment pathways.

Presence of a WMH led subtype in an ADNI cohort that excludes those with increased vascular burden at screening (Hachinski score > 4), suggests cerebrovascular disease impact may be increased in community‐based populations. Markers like WMH provide important information about individuals’ disease pathways and must be considered.

### 4.6 Limitations

This version of SuStaIn provides temporal ordering, but not relative timings between events. A more recent development of SuStaIn has included this feature^56^ and should be explored.

This work assumes amyloid abnormality occurs prior to other biomarkers. Some early events in subtypes may occur prior to amyloid abnormality, like WMH volumes in the WMH led subtype.

*Z* score SuStaIn assumes a linear pattern, meaning individuals would be expected to follow their subtype longitudinally. This cannot currently be considered, although previous work has validated SuStaIn longitudinally.^12^, ^13^, ^14^

Sample sizes in subtypes beyond event stages ten are small (Figure S4) and this makes later stage interpretation more difficult.

The use of a different *z* score normalization group would influence subtype identification. CSF amyloid‐negative, *APOE* ε4 non‐carrier, diagnostically stable CN individuals were used to produce *z* scores so as to have more confidence this group represented healthy aging.

The existence of a Memory led group is unsurprising as the normalization group is cognitively unimpaired. Post hoc exploration of SuStaIn without LM (Figures S8, S9, S10 in supporting information) did not overly alter derived subtypes.

## 5 CONCLUSION

Four amyloid‐positive subtypes were derived using data‐driven *z* score SuStaIn. Two of these subtypes (Memory led and p‐tau led), were likely subtly different populations than those on the AD pathway: one being more indicative of an AD‐typical pathway (p‐tau led), and the other with both AD and some potential co‐pathology (Memory led). Interestingly, an additional subtype of individuals with much higher presumed vascular pathology (WMH led) was also present. An unsubtyped group was amyloid positive and may represent a very early AD group with amyloid abnormality. This work has shown the heterogeneity in a cohort of individuals that may be assumed homogeneous. Our results show the importance of using data‐driven techniques that find heterogeneity in disease and has stressed the influence of presumed cerebrovascular pathology and other co‐pathologies in those who are amyloid positive.

## CONFLICT OF INTEREST STATEMENT

L. Prosser reports no disclosures additional to those that support the DRC. C. H. Sudre reports no disclosures additional to those that support the DRC. N. P. Oxtoby consults for Queen Square Analytics Limited (UK). A. L. Young was supported by the Wellcome Trust (227341/Z/23/Z). I. B. Malone is supported by grants to his institution from NIH and is an employee of the DRC which is supported by Alzheimer's Research UK, Brain Research Trust, and The Wolfson Foundation. E. M. Manning reports no disclosures additional to those that support the DRC. H. Pemberton reports no disclosures additional to those that support the DRC. P. Walsh reports no disclosures additional to those that support the DRC. F. Barkhof supported by the NIHR biomedical research centre at UCLH. F. Barkhof is part of the Steering committee or data safety monitoring board member for Biogen, Merck, Eisai, and Prothena; is an advisory board member for Combinostics and Scottish Brain Sciences; a consultant for Roche, Celltrion, Rewind Therapeutics, Merck, and Bracco; has research agreements with ADDI, Merck, Biogen, GE Healthcare, and Roche; and is a co‐founder and shareholder of Queen Square Analytics LTD. G. J. Biessels reports no additional disclosures. D. M. Cash reports no disclosures additional to those that support the DRC. J. Barnes reports no disclosures additional to those that support the DRC. Author disclosures are available in the supporting information.

## CONSENT STATEMENT

For ADNI, protocol and informed consent forms were approved by the institutional review board at each participating site.

## Supporting information



Supporting Information

Supporting Information

## References

[1] Jack Jr CR , Bennett DA , Blennow K , et al. NIA‐AA Research Framework: toward a biological definition of Alzheimer's disease. Alzheimer's Dement. 2018;14(4):535‐562. doi:10.1016/j.jalz.2018.02.018 29653606 PMC5958625

[2] Selkoe DJ , Hardy J . The amyloid hypothesis of Alzheimer's disease at 25 years. EMBO Mol Med. 2016;8(6):595‐608. 27025652 10.15252/emmm.201606210 PMC4888851

[3] Hardy JA , Higgins GA . Alzheimer's disease: the amyloid cascade hypothesis. Science (1979). 1992;256(5054):184‐186. 10.1126/science.1566067 1566067

[4] Fiford CM , Sudre CH , Young AL , et al. Presumed small vessel disease, imaging and cognition markers in the Alzheimer's Disease Neuroimaging Initiative. Brain Commun. 2021;3(4):fcab226. 34661106 10.1093/braincomms/fcab226 PMC8514859

[5] Habes M , Grothe MJ , Tunc B , McMillan C , Wolk DA , Davatzikos C . Disentangling heterogeneity in Alzheimer's disease and related dementias using data‐driven methods. Biol Psychiatry. 2020;88(1):70‐82. doi:10.1016/J.BIOPSYCH.2020.01.016 32201044 PMC7305953

[6] Salloway S , Lee E , Papka M , et al. TRAILBLAZER‐ALZ 4: topline study results directly comparing donanemab to aducanumab on amyloid lowering in early, symptomatic Alzheimer's disease (S26.009). Neurology. 2023;100(17):3126. doi:10.1212/WNL.0000000000203040. Supplement 2.

[7] Ferreira D , Mohanty R , Murray ME , Nordberg A , Kantarci K , Westman E . The hippocampal sparing subtype of Alzheimer's disease assessed in neuropathology and in vivo tau positron emission tomography: a systematic review. Acta Neuropathol Commun. 2022;10(1):1‐19. doi:10.1186/S40478-022-01471-Z/FIGURES/3 36376963 PMC9664780

[8] Jansen WJ , Ossenkoppele R , Knol DL , et al. Prevalence of cerebral amyloid pathology in persons without dementia: a meta‐analysis. JAMA—Journal of the American Medical Association. 2015;313(19):1924‐1938. doi:10.1001/JAMA.2015.4668 PMC4486209 25988462

[9] Prosser L , MacDougall A , Sudre CH , et al. Predicting cognitive decline in older adults using baseline metrics of AD pathologies, cerebrovascular disease, and neurodegeneration. Neurology. 2023;100(8):e834‐e845. doi:10.1212/WNL.0000000000201572 36357185 PMC9984210

[10] Roseborough A , Ramirez J , Black SE , Edwards JD . Associations between amyloid β and white matter hyperintensities: a systematic review. Alzheimer's and Dementia. 2017;13(10):1154‐1167. doi:10.1016/J.JALZ.2017.01.026 28322203

[11] Young AL , Marinescu RV , Oxtoby NP , et al. Uncovering the heterogeneity and temporal complexity of neurodegenerative diseases with subtype and stage inference. Nature Communications 2018 9:1. 2018;9(1):1‐16. doi:10.1038/s41467-018-05892-0 PMC6189176 30323170

[12] Zhou C , Wang L , Cheng W , et al. Two distinct trajectories of clinical and neurodegeneration events in Parkinson's disease. npj Parkinson's Disease 2023 9:1. 2023;9(1):1‐11. doi:10.1038/s41531-023-00556-3 PMC10344958 37443179

[13] Young AL , Vogel JW , Robinson JL , et al. Data‐driven neuropathological staging and subtyping of TDP‐43 proteinopathies. Brain. 2023;146(7):2975‐2988. doi:10.1093/BRAIN/AWAD145 37150879 PMC10317181

[14] Aksman LM , Oxtoby NP , Scelsi MA , et al. A data‐driven study of Alzheimer's disease related amyloid and tau pathology progression. Brain. 2023;146. doi:10.1093/brain/awad232 PMC10690020 37433038

[15] Sudre CH , Cardoso MJ , Bouvy WH , Biessels GJ , Barnes J , Ourselin S . Bayesian model selection for pathological neuroimaging data applied to white matter lesion segmentation. IEEE Trans Med Imaging. 2015;34(10):2079‐2102. 25850086 10.1109/TMI.2015.2419072

[16] Fiford CM , Sudre CH , Pemberton H , et al. Automated white matter hyperintensity segmentation using bayesian model selection: assessment and correlations with cognitive change. Neuroinformatics. 2020:1‐21. Published online. 32062817 10.1007/s12021-019-09439-6 PMC7338814

[17] Gregoire SM , Chaudhary UJ , Brown MM , et al. The Microbleed Anatomical Rating Scale (MARS): reliability of a tool to map brain microbleeds. Neurology. 2009;73(21):1759‐1766. 19933977 10.1212/WNL.0b013e3181c34a7d

[18] Leung KK , Barnes J , Modat M , et al. Brain MAPS: an automated, accurate and robust brain extraction technique using a template library. Neuroimage. 2011;55(3):1091‐1108. 21195780 10.1016/j.neuroimage.2010.12.067 PMC3554789

[19] Freeborough PA , Fox NC . The boundary shift integral: an accurate and robust measure of cerebral volume changes from registered repeat MRI. IEEE Trans Med Imaging. 1997;16(5):623‐629. 9368118 10.1109/42.640753

[20] Cardoso MJ , Leung K , Modat M , et al. STEPS: similarity and truth estimation for propagated segmentations and its application to hippocampal segmentation and brain parcelation. Med Image Anal. 2013;17(6):671‐684. 23510558 10.1016/j.media.2013.02.006

[21] Cardoso MJ , Modat M , Wolz R , et al. Geodesic information flows: spatially‐variant graphs and their application to segmentation and fusion. IEEE Trans Med Imaging. 2015;34(9):1976‐1988. doi:10.1109/TMI.2015.2418298 25879909

[22] Sánchez‐Cubillo I , Periáñez JA , Adrover‐Roig D , et al. Construct validity of the Trail Making Test: role of task‐switching, working memory, inhibition/interference control, and visuomotor abilities. J Int Neuropsychol Soc. 2009;15(3):438‐450. doi:10.1017/S1355617709090626 19402930

[23] Gisslén M , Price RW , Andreasson U , et al. Plasma concentration of the neurofilament light protein (NFL) is a biomarker of CNS injury in HIV infection: a cross‐sectional study. EBioMedicine. 2016;3:135‐140. doi:10.1016/j.ebiom.2015.11.036 26870824 PMC4739412

[24] O'Brien PC , Dyck PJ . Procedures for setting normal values. Neurology. 1995;45(1):17‐23. doi:10.1212/WNL.45.1.17/ASSET/357829CB-5F98-4B38-AC1A-AB319A7FC6D2/ASSETS/WNL.45.1.17.FP.PNG 7824110

[25] Fonteijn HM , Modat M , Clarkson MJ , et al. An event‐based model for disease progression and its application in familial Alzheimer's disease and Huntington's disease. Neuroimage. 2012;60(3):1880‐1889. doi:10.1016/J.NEUROIMAGE.2012.01.062 22281676

[26] Young AL , Oxtoby NP , Daga P , et al. A data‐driven model of biomarker changes in sporadic Alzheimer's disease. Brain. 2014;137(9):2564‐2577. doi:10.1093/BRAIN/AWU176. Pt. 25012224 PMC4132648

[27] Toledo JB , Arnold SE , Raible K , et al. Contribution of cerebrovascular disease in autopsy confirmed neurodegenerative disease cases in the National Alzheimer's Coordinating Centre. Brain. 2013;136(9):2697‐2706. doi:10.1093/BRAIN/AWT188 23842566 PMC3858112

[28] Santos CY , Snyder PJ , Wu WC , Zhang M , Echeverria A , Alber J . Pathophysiologic relationship between Alzheimer's disease, cerebrovascular disease, and cardiovascular risk: a review and synthesis. Alzheimer's & Dementia: Diagnosis, Assessment & Disease Monitoring. 2017;7:69‐87. doi:10.1016/J.DADM.2017.01.005 PMC5328683 28275702

[29] Huijbers W , Mormino EC , Schultz AP , et al. Amyloid‐β deposition in mild cognitive impairment is associated with increased hippocampal activity, atrophy and clinical progression. Brain. 2015;138(4):1023‐1035. doi:10.1093/BRAIN/AWV007 25678559 PMC4438387

[30] Henneman WJP , Sluimer JD , Barnes J , et al. Hippocampal atrophy rates in Alzheimer disease: added value over whole brain volume measures. Neurology. 2009;72(11):999‐1007. 19289740 10.1212/01.wnl.0000344568.09360.31 PMC2821835

[31] Duff K . Amnestic MCI in ADNI: maybe not enough memory impairment?. Neurology. 2021;97(12):595‐596. 34341152 10.1212/WNL.0000000000012587 PMC8480486

[32] Kalaria R . Similarities between Alzheimer's disease and vascular dementia. J Neurol Sci. 2002;203:29‐34. 12417353 10.1016/s0022-510x(02)00256-3

[33] De Reuck J , Deramecourt V , Cordonnier C , et al. The incidence of post‐mortem neurodegenerative and cerebrovascular pathology in mixed dementia. J Neurol Sci. 2016;366:164‐166. 27288798 10.1016/j.jns.2016.05.021

[34] Leiros BG , Mendez LIP , Huerta MVZ , et al. Prevalence and concordance between the clinical and the post‐mortem diagnosis of dementia in a psychogeriatric clinic. Neurología (English Edition). 2018;33(1):13‐17. 10.1016/j.nrl.2016.04.011 27328891

[35] Jellinger KA . The enigma of mixed dementia. Alzheimer's & Dementia. 2007;3(1):40‐53. doi:10.1016/J.JALZ.2006.09.002 19595916

[36] Mattson MP . Late‐onset dementia: a mosaic of prototypical pathologies modifiable by diet and lifestyle. npj Aging and Mechanisms of Disease 2015 1:1. 2015;1(1):1‐11. doi:10.1038/npjamd.2015.3 PMC5478237 28642821

[37] Zhuang FJ , Chen Y , He WB , Cai ZY . Prevalence of white matter hyperintensities increases with age. Neural Regen Res. 2018;13(12):2141. doi:10.4103/1673-5374.241465 30323144 PMC6199954

[38] Meeker KL , Butt OH , Gordon BA , et al. Cerebrospinal fluid neurofilament light chain is a marker of aging and white matter damage. Neurobiol Dis. 2022;166:105662. doi:10.1016/J.NBD.2022.105662 35167933 PMC9112943

[39] van de Pol L , Gertz HJ , Scheltens P , Wolf H . Hippocampal atrophy in subcortical vascular dementia. Neurodegener Dis. 2011;8(6):465‐469. doi:10.1159/000326695 21613775

[40] Freeze WM , Jacobs HIL , Gronenschild EH , et al. White matter hyperintensities potentiate hippocampal volume reduction in non‐demented older individuals with abnormal amyloid‐β. Journal of Alzheimer's Disease. 2017;55(1):333‐342. doi:10.3233/JAD-160474 27662299

[41] De Jong D , Jansen RWMM , Kremer BPH , Verbeek MM . Cerebrospinal fluid amyloid β42/phosphorylated tau ratio discriminates between Alzheimer's disease and vascular dementia. J Gerontol A Biol Sci Med Sci. 2006;61(7):755‐758. 16870640 10.1093/gerona/61.7.755

[42] Mukaetova‐Ladinska EB , Abdel‐All Z , Mugica S , et al. Tau proteins in the temporal and frontal cortices in patients with vascular dementia. J Neuropathol Exp Neurol. 2015. Published online. Accessed June 1, 2023. https://academic.oup.com/jnen/article/74/2/148/2614309 10.1097/NEN.0000000000000157 25575131

[43] Vemuri P , Lesnick TG , Przybelski SA , et al. Vascular and amyloid pathologies are independent predictors of cognitive decline in normal elderly. Brain. 2015;138(3):761‐771. doi:10.1093/BRAIN/AWU393 25595145 PMC4339775

[44] Landau SM , Mintun MA , Joshi AD , et al. Amyloid deposition, hypometabolism, and longitudinal cognitive decline. Ann Neurol. 2012;72(4):578‐586. doi:10.1002/ANA.23650 23109153 PMC3786871

[45] Debette S , Markus HS . The clinical importance of white matter hyperintensities on brain magnetic resonance imaging: systematic review and meta‐analysis. BMJ. 2010:341. 10.1136/bmj.c3666 PMC2910261 20660506

[46] Jack Jr CR , Knopman DS , Jagust WJ , et al. Update on hypothetical model of Alzheimer's disease biomarkers. Lancet Neurol. 2013;12(2):207. 23332364 10.1016/S1474-4422(12)70291-0 PMC3622225

[47] Salvadó G , Milà‐Alomà M , Shekari M , et al. Cerebral amyloid‐β load is associated with neurodegeneration and gliosis: mediation by p‐tau and interactions with risk factors early in the Alzheimer's continuum. Alzheimer's & Dementia. 2021;17(5):788‐800. 10.1002/alz.12245 PMC8252618 33663013

[48] Braak H , Braak E . Neuropathological stageing of Alzheimer‐related changes. Acta Neuropathol. 1991;82(4):239‐259. 1759558 10.1007/BF00308809

[49] van de Pol LA , Hensel A , Barkhof F , Gertz HJ , Scheltens P , van der Flier WM . Hippocampal atrophy in Alzheimer disease: age matters. Neurology. 2006;66(2):236‐238. doi:10.1212/01.wnl.0000194240.47892.4d 16434661

[50] Garnier‐Crussard A , Bougacha S , Wirth M , et al. White matter hyperintensities across the adult lifespan: relation to age, Aβ load, and cognition. Alzheimers Res Ther. 2020;12(1):1‐11. doi:10.1186/S13195-020-00669-4/FIGURES/3 PMC7545576 33032654

[51] Larsson SC , Markus HS . Does treating vascular risk factors prevent dementia and Alzheimer's disease? A systematic review and meta‐analysis. Journal of Alzheimer's Disease. 2018;64(2):657‐668. doi:10.3233/JAD-180288 29914039

[52] Ligthart SA , van Charante EPM , van Gool WA , Richard E . Treatment of cardiovascular risk factors to prevent cognitive decline and dementia: a systematic review. Vasc Health Risk Manag. 2010;6(1):775‐785. doi:10.2147/VHRM.S7343 20859546 PMC2941788

[53] Fagan AM . What does it mean to be ‘amyloid‐positive’?. Brain. 2015;138(3):514. doi:10.1093/BRAIN/AWU387 25713403 PMC4408431

[54] Mattsson N , Insel PS , Donohue M , et al. Independent information from cerebrospinal fluid amyloid‐β and florbetapir imaging in Alzheimer's disease. Brain. 2015;138(3):772. doi:10.1093/BRAIN/AWU367 25541191 PMC4339769

[55] Liu CC , Zhao N , Fu Y , et al. ApoE4 accelerates early seeding of amyloid pathology in brief. Neuron. 2017;96:1024‐1032. doi:10.1016/j.neuron.2017.11.013. e3. 29216449 PMC5948105

[56] Young AL , Aksman LM , Alexander DC , Wijeratne PA . Subtype and stage inference with timescales. LNCS. 2023;13939:15‐26. doi:10.1007/978-3-031-34048-2_2

