# Supplementary material for: Biomarker pathway heterogeneity of amyloid‐positive individuals
Source: Alzheimers Dement. 2024 Oct 17;20(12):8503–15. doi: 10.1002/alz.14287 (PMC11667528; doi:10.1002/alz.14287)
Supplement: Supplementary file 1 — Supporting Information [file ALZ-20-8503-s001.docx]

**Supplementary materials**

The histogram of amyloid positive z-scores for each marker is reported below in supplementary figure 1. z-scores 1,2, and 3 are representative of approximately 75% of amyloid-positive cases, with the maximum number of z-scores (5) resting towards the tail end of most of the sample.


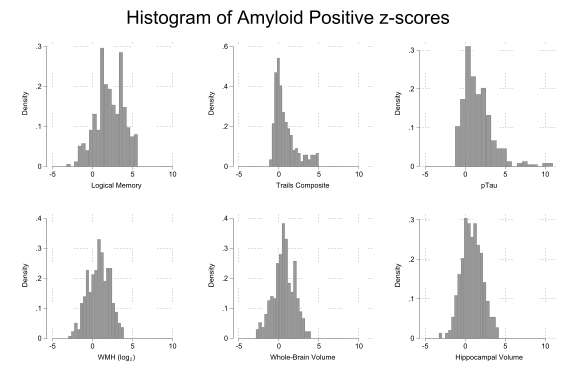


**Supplementary Figure 1.** Histogram of amyloid positive z-scores. Histograms represent frequency of z-scores per marker included in the SuStaIn model.

*Abbreviations:* pTau = phosphorylated tau; WMH = white matter hyperintensities.

The MCMC trace using five potential subtypes is shown below in supplementary figure 2. The overlap in log likelihood for additional subtypes past three suggest more subtypes would not provide additional information around distinctly heterogeneous groups within those amyloid positive. The CVIC supplementary figure 3. further suggests that the best balance is found using three subtypes.


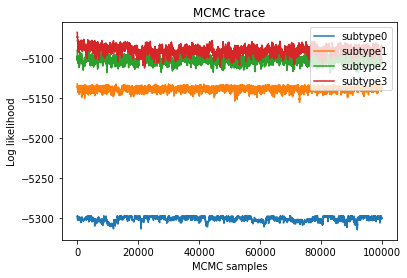


**Supplementary Figure 2.** MCMC trace when using four possible subtypes.

*Abbreviations:* MCMC = marcov chain monte carlo


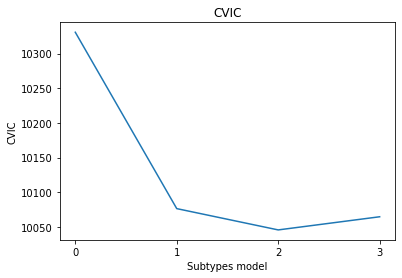


**Supplementary Figure 3.** CVIC for four possible subtypes.

*Abbreviations:* CVIC = cross-validation information criterion

Below we report histogram plots for the individuals present at each stage within the three subtypes. Lower sample sizes at later stages (above 10) reduce stage certainty, and reflect lower biomarker certainty at these later stages.


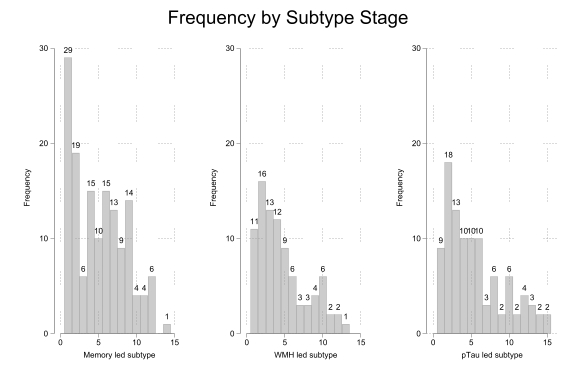


**Supplementary figure 4.** Frequency plot of individuals per subtype stage. Bars representing subjects included in each stage.

*Abbreviations:* pTau = phosphorylated tau.

Below we plot subtype probabilities over SuStaIn stages to ensure no crossover events are present. A crossover event would be recognized with probabilities resting below 50%, suggesting that subjects in this stage could be classified in another subtype. Probabilities are above 60% for all stages, apart from stage 14 of the memory led subtype, which rests at around 50%. This suggests that some crossover may be present at this stage.


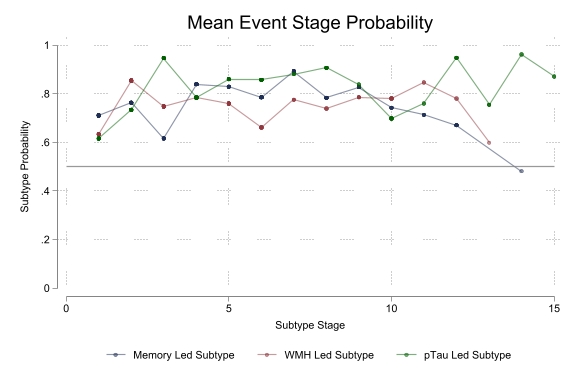


**Supplementary Figure 5.** Probability plots for each subtype over stage.

A flow diagram of sample selection is reported below in supplementary figure 6. From an initial sample of 649, a total of 211 were excluded.

**
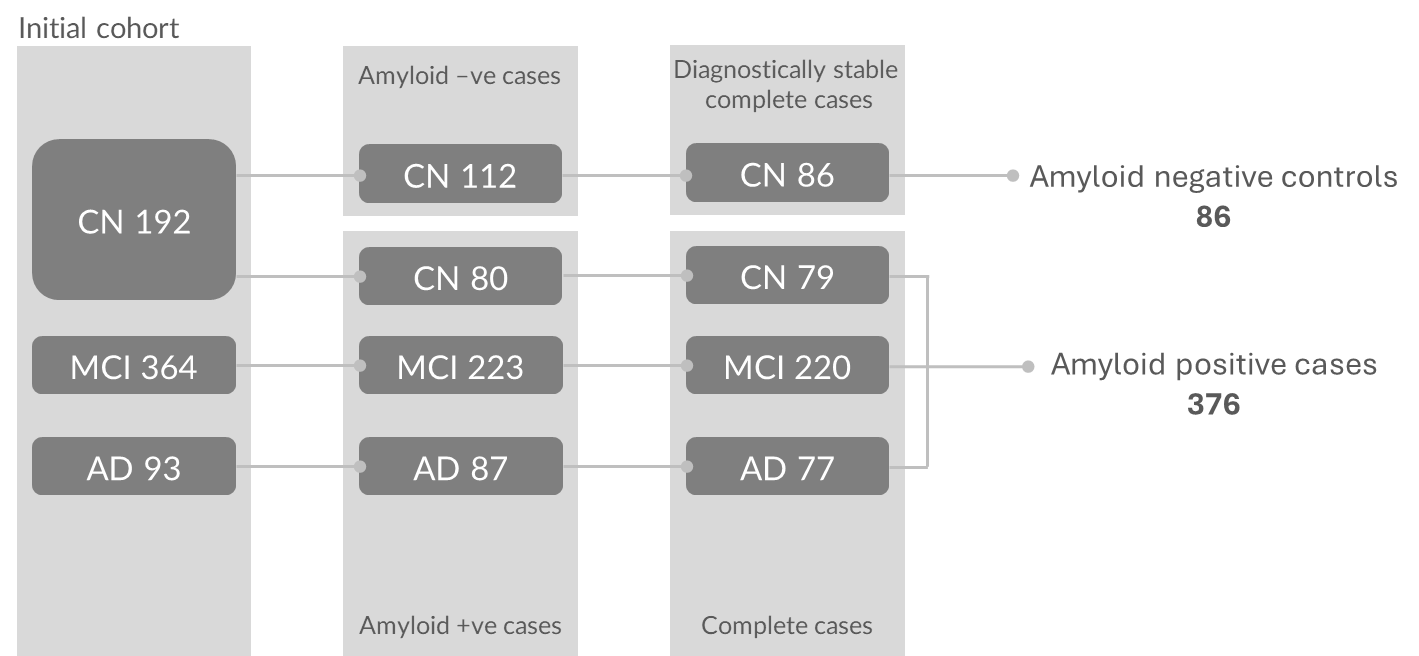
**

***Supplementary Figure 6.*** Flow chart of initial cohort, and those used in the current work. Completed cases means those with all marker data. There were no missing data for amyloid-negative controls. Direction of flow goes from left to right.

*Abbreviations*: -ve = negative; +ve = positive

Pairwise correlation coefficients and significance values that support figure 2 are reported below. There is evidence to suggest a significant difference for each marker over event stage within each subtype (*p* < .03; all tests), although the is no evidence to suggest a difference in WMH volumes over stage in the WMH led subtype. The presence of a ceiling effect in TMT B (time taken to complete tasking exceeding 300 seconds), may artificially reduce the correlation coefficient in the pairwise correlation below.

| Supplementary table 1: Accompanying pairwise correlation coefficient and *p* value for figure 2 | | | | |
| --- | --- | --- | --- | --- |
|  | Subtype | | | |
|  | Memory Led | WMH Led | pTau Led |  |
| N# | 145 | 88 | 100 |  |
| First assessment LM | 0.7; *p* < .001 | 0.8; *p* < .001 | 0.7; *p* < .001 |  |
| First assessment composite TMT^a^ | 0.5; *p* < .001 | 0.6; *p* < .001 | 0.5; *p* < .001 |  |
| CSF pTau-181 | 0.5; *p* < .001 | 0.2; *p* = .03 | 0.6; *p* < .001 |  |
| WMH volume^b^ | 0.3; *p* < .001 | 0.02; *p* = 0.9 | 0.4; *p* < .001 |  |
| Whole-brain volume^b^ | 0.7; *p* < .001 | 0.5; *p <* 0.01 | 0.5; *p* < .001 |  |
| Hippocampal Volume^b^ | 0.7; *p* < .001 | 0.5; *p* < .001 | 0.4; *p* < .001 |  |
| *Note:* Pairwise correlation coefficient, assessing relationship between biomarker and SuStaIn stage within subtypes. Analysis performed using z-scores. Semi-partial correlation is reported for TMT, WMH volume, Whole-brain volume, and hippocampal volume.  ^a^ Adjusted for presence of ceiling effect in TMT B (time to complete > 300 seconds).  ^b^ Adjusted for TIV.  *Abbreviations:* N# = number; LM = logical memory; TMT = trail making task; pTau = phosphorylated tau; WMH = white matter hyperintensities | | | | |

Further explanation for missing diagnostic data of the subtypes is reported below in table 2. On average there were more MCI individuals with missing data compared to CN. Of MCI individuals the main reason for missingness was lack of follow-up past twelve months.

| **Supplementary table 2:** diagnostic data missingness breakdown | | | | | |
| --- | --- | --- | --- | --- | --- |
|  | Unsubtyped | Subtype | | |  |
|  |  | One | Two | Three |  |
| Missing progression data CN, N# (%) | 3 (13) | 1 (14) | 3 (10) | 4 (20) |  |
| *Limited data, non-progressors* |  |  |  |  |  |
| Final available data at month six | 2 | - | - | 1 |  |
| Final available data at month twelve | 1 | - | 2 | 2 |  |
| Only available future data at month 36 | - | 1 | - | - |  |
| *Limited data, progressors (to MCI)* |  |  |  |  |  |
| Final available data at month six | - | - | 1 | 1 |  |
| No diagnostic information after baseline | - | - | - | 1 |  |
|  |  |  |  |  |  |
| Missing progression data MCI, N# (%) | 4 (20) | 16 (17) | 10 (20) | 8 (14) |  |
| *Limited data, non-progressors* |  |  |  |  |  |
| Final available data at month six | - | 3 | 1 | - |  |
| Final available data at month twelve | 3 | 9 | 5 | 5 |  |
| Only available future data at month 36 |  | 2 | - | 3 |  |
| Final available future data at month 72 | - | - | 1 | - |  |
| *Limited data, progressors (to MCI)* |  |  |  |  |  |
| Only available future data at month 36 | - | 2 | 1 | - |  |
| Only available future data at month 72 | - | - | 1 | - |  |
| No diagnostic information after baseline | 1 | - | 1 | - |  |
| *Note:* Further breakdown of missing diagnostic data for each subtype (and unsubtyped), by baseline diagnosis for those remaining stable or progressing diagnostically.  *Abbreviations:* CN = cognitively unimpaired; MCI = mild cognitive impairment; N# = number | | | | |  |

Violin plots of amyloid over each subtype, and the unsubtyped group are displayed below in supplementary figure 7. As expected, the gaussian spread of the unsubtyped does skew closer to the cut-point compared to the other subtypes, although there is still spread of those with increased amyloid abnormality.


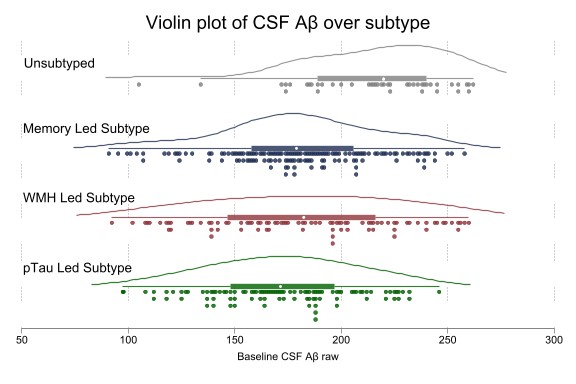


**Supplementary figure 7.** Violin plot of raw CSF amyloid over subtype.

*Abbreviations:* Aβ = amyloid (beta 1-42)

A post-hoc exploration of SuStaIn was produced to explore the bias of including LM (a general measure of cognitive impairment), in the SuStaIn model. Positional variance diagrams are majorly unchanged (supplementary figure 8), with a more hippocampal led subtype (previous named memory led), a WMH led subtype, and a pTau led subtype. The hippocampal led subtype does have on average the lowest LM score compared to the other subtypes (*p* = 0.01), suggesting this subtype is likely more cognitively impaired. A frequency by subtype stage is also plotted to help make inferences about uncertainty in the model (supplementary figure 9), with dotted lines added to the positional variance diagrams where sample size is report at 2 of less for two consecutive stages. A biomarkers per subtype stage is also produced (supplementary figure 10).


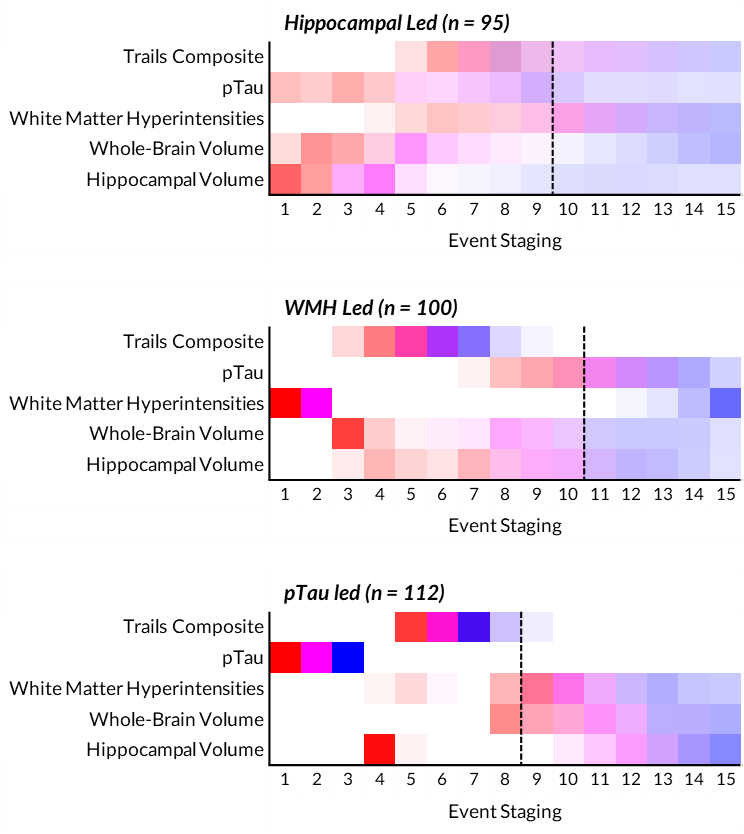


***Supplementary Figure 8*.** Positional variance diagrams of SuStaIn derived subtypes, without using LM. SuStaIn subtyping depicting biomarker event staging by ordering of standardized scores (one z-score [Red], two z-scores [magenta], three z-scores [blue]), with colors between these scales highlight uncertainty in event staging. WMH used log_2_. A dashed line for each subtype is included to represent higher uncertainty in the diagrams, where number of subjects is 2 or less for two consecutive stages.

*Abbreviations*: SuStaIn = Subtype and Stage Inference; WMH = white matter hyperintensities

***Supplementary figure 9.*** Frequency plot of individuals per subtype stage.

*Abbreviations:* pTau = phosphorylated tau.

***Supplementary figure 10.*** Biomarkers by SuStaIn stage, across subtype. Individual volumes for subtypes for each stage across subtype are plotted, with a line of best fit.

*Abbreviations:* pTau = phosphorylated tau; WMH = white matter hyperintensities
